# Post-CDK4/6 Inhibitor Therapeutic Approaches in Hormone Receptor-Positive, HER2-Negative Metastatic Breast Cancer: Current Evidence and Emerging Strategies—A Narrative Review

**DOI:** 10.3390/diagnostics16121790

**Published:** 2026-06-10

**Authors:** Humaid O. Al-Shamsi, Nadia Abdelwahed, Siddig Ibrahim Abdelwahab, Mawada Hussein, Amin Abyad, Saeed Rafii, Hassan Jaafar, Sonia Otsmane, Dima Abdul Jabbar, Hala Abdellatif, Faryal Iqbal, Mudhasir Ahmad, Hampig Kourie, Kefah Mokbel

**Affiliations:** 1 Burjeel Cancer Institute, Burjeel Medical City, Abu Dhabi P.O. Box 92510, United Arab Emirates; nadia.abdelwahed@burjeelmedicalcity.com (N.A.); mawada.mohamed@burjeelmedicalcity.com (M.H.); amin.abyad@burjeelmedicalcity.com (A.A.); hassan.jaafar@burjeelmedicalcity.com (H.J.); sonia.otsmane@burjeelmedicalcity.com (S.O.); dima.jabbar@burjeelmedicalcity.com (D.A.J.); hala.abdellatif@burjeelmedicalcity.com (H.A.); faryal.iqbal@burjeelmedicalcity.com (F.I.); drmudhasir.a@lifecarehospital.ae (M.A.); 2 Department of Medical Oncology, Dana-Farber Cancer Institute, Harvard Medical School, Boston, MA 02215, USA; 3 College of Medicine, Ras Al Khaimah Medical and Health Sciences University, Al Juwais, Al Qusaidat, Ras Al Khaimah P.O. Box 11172, United Arab Emirates; 4 College of Medicine, Gulf Medical University, Ajman P.O. Box 4184, United Arab Emirates; 5 Emirates Oncology Society, Dubai P.O Box 6600, United Arab Emirates; 6 Health Research Centre, Jazan University, Jazan 45142, Saudi Arabia; sadiqa@jazanu.edu.sa; 7 Department of Oncology, Mediclinic City Hospital, Dubai P.O. Box 505004, United Arab Emirates; saeed.rafii@mediclinic.ae; 8 Hematology-Oncology Department, Faculty of Medicine, Saint Joseph University, Beirut P.O. Box 11-5076, Lebanon; hampig.kourie@usj.edu.lb; 9 The London Breast Institute, Princess Grace Hospital, London W1U 5NY, UK; kefahmokbel2@gmail.com

**Keywords:** CDK4/6 inhibitors, ESR1 mutations, ctDNA, elacestrant, inavolisib, capivasertib

## Abstract

**Background**: Therapeutic resistance following cyclin-dependent kinase 4/6 inhibitor (CDK4/6i) plus endocrine therapy (ET) represents a key unmet need in hormone receptor-positive, human epidermal growth factor receptor 2-negative (HR+/HER2−) metastatic breast cancer (mBC). Treatment paradigms have advanced from non-targeted options, such as fulvestrant monotherapy or everolimus-based combinations, to precision medicine strategies, including inhibitors of the PI3K/AKT pathway, oral selective estrogen receptor degraders (SERDs), and novel ER-modulating agents, often guided by biomarkers and molecular surveillance. **Methods**: This narrative review synthesizes evidence from randomized clinical trials, real-world studies, and biomarker-driven analyses published from 2010 to 2026, with emphasis on next-generation sequencing (NGS)-guided genomic profiling, targeted pathway therapies, and circulating tumor DNA (ctDNA)-based proactive interventions in the post-CDK4/6i setting. This review was conducted and reported in accordance with the SANRA recommendations for narrative reviews. **Results**: Early second-line standards, including fulvestrant and alpelisib for PIK3CA-mutated tumors, established the basis for biomarker-guided treatment in hormone receptor–positive, HER2-negative metastatic breast cancer. With the widespread use of CDK4/6 inhibitors in the first-line setting, the optimal post-progression strategy has shifted toward molecularly selected combination approaches rather than single-agent endocrine therapy, as endocrine monotherapy has shown limited efficacy in acquired resistance. Multiple randomized studies have demonstrated that adding targeted agents to endocrine therapy improves progression-free survival compared with hormonal therapy alone, supporting combination regimens as the preferred strategy after CDK4/6 inhibitor progression, except in carefully selected patients with low disease burden, indolent biology, or frailty where tolerability is a major concern. Precision-based trials have further refined this approach. Elacestrant improved progression-free survival in ESR1-mutated disease in the EMERALD trial, capivasertib plus fulvestrant demonstrated significant benefit in tumors harboring AKT/PIK3CA/PTEN pathway alterations in CAPItello-291, and inavolisib plus palbociclib and fulvestrant achieved both progression-free and overall survival improvement in PIK3CA-mutated patients with early relapse in INAVO120. Real-world analyses further support the effectiveness of these biomarker-directed strategies across diverse clinical subgroups. Comprehensive genomic profiling has identified multiple resistance mechanisms, including ESR1 mutations, PI3K/AKT/mTOR pathway activation, RB1 loss, and FGFR alterations, which may co-occur and reduce sensitivity to endocrine monotherapy. While ESR1 and PI3K pathway alterations now guide approved therapies, FGFR alterations remain investigational targets, with ongoing trials evaluating selective FGFR inhibitors. Proactive switching approaches evaluated in SERENA-6 and PADA-1 demonstrate that serial circulating tumor DNA (ctDNA) monitoring can detect emergent ESR1 mutations before radiographic progression, providing a clinically actionable lead time for early therapeutic modification and extending endocrine-based disease control by approximately 5 to 7 months. **Conclusions**: Post-CDK4/6i management increasingly relies on NGS-guided precision approaches, integrating pathway-specific therapies and ctDNA surveillance to tailor sequencing based on resistance profiles, prior ET response, and tumor heterogeneity. Future investigations into novel ER degraders and multi-targeted combinations hold potential to further optimize algorithms, extend non-chemotherapy options, and enhance survival in HR+/HER2− mBC.

## 1. Introduction

Hormone receptor-positive, human epidermal growth factor receptor 2-negative metastatic breast cancer (HR+/HER2− mBC) accounts for approximately 60 to 70% of advanced breast cancers and represents the largest biologic subtype requiring chronic systemic therapy [1]. The incorporation of cyclin-dependent kinase 4/6 inhibitors (CDK4/6i)—palbociclib, ribociclib, and abemaciclib—into first-line endocrine therapy has extended median progression-free survival (PFS) to approximately 24 to 28 months in contemporary cohorts, with overall survival (OS) frequently exceeding 38 to 58 months in selected populations [2,3,4]. These advances have transformed HR+/HER2− mBC into a molecularly stratified, long-trajectory disease.

Resistance after CDK4/6 inhibitor exposure is biologically heterogeneous and may involve acquired ESR1 mutations, PI3K/AKT/PTEN pathway activation, RB1 loss, FGFR alterations, and other cell-cycle escape mechanisms [5]. These alterations may coexist, supporting the need for longitudinal molecular monitoring and biomarker-directed therapeutic sequencing [6].

ESR1 mutations, which are an emerging or acquired mechanism of resistance, arise in approximately 30 to 48% of tumors following prolonged endocrine exposure, while intrinsic PI3K/AKT/PTEN pathway alterations occur in 40 to 50% (PIK3CA ~30 to 40%, AKT1 ~4 to 5%, PTEN loss ~5%) [7,8,9,10]. Additional mechanisms, which can be both intrinsic and acquired, include RB1 loss (2 to 9%), FGFR amplifications (15 to 41%), and cyclin E amplification. Whole-exome sequencing of CDK4/6i-exposed tumors has identified resistance alterations in 66% of cases, with nearly one-third harboring multiple concurrent drivers, underscoring the polyclonal and adaptive nature of post-progression disease [5].

Progression following first-line CDK4/6i therapy necessitates biomarker-directed therapeutic sequencing. In PIK3CA-mutated disease, alpelisib plus fulvestrant remains an established standard of care based on SOLAR-1, while inavolisib-based triplet therapy has demonstrated both progression-free and OS benefit in endocrine-resistant populations. For tumors harboring AKT, PIK3CA, or PTEN alterations, capivasertib plus fulvestrant provides pathway-directed benefit [4,6,11,12,13,14,15]. In ESR1-mutated disease without dominant PI3K pathway activation, oral selective estrogen receptor degraders such as elacestrant represent evidence-supported options [7,15,16,17]. PARP inhibitors are indicated in germline BRCA1/2-mutated disease. Emerging strategies, including giredestrant-based combinations and the gedatolisib triplet evaluated in VIKTORIA-1 for PIK3CA-wild-type tumors, may further expand second-line options pending regulatory maturation.

Three critical challenges define the post-CDK4/6i era: (1) prolonging endocrine sensitivity while delaying chemotherapy exposure; (2) optimizing sequencing in tumors with co-occurring ESR1 and PI3K pathway alterations (present in 20 to 40% of cases); and (3) integrating proactive molecular surveillance strategies to intervene before radiographic progression. This review synthesizes evidence across three clinically actionable domains: (i) ctDNA-guided proactive switching paradigms, (ii) real-world performance of newly approved agents relative to trial benchmarks, and (iii) molecular decision frameworks for complex, co-altered disease. The focus is restricted to endocrine-based strategies, PI3K/AKT/mTOR inhibitors, and next-generation estrogen receptor degraders, providing a precision-oriented roadmap for post-CDK4/6i therapeutic sequencing in HR+/HER2− mBC. Regulatory approvals referenced reflect U.S. FDA status as of early 2026.

### Generational Evolution of CDK4/6 and PI3K Inhibitors

The development of CDK4/6 and PI3K inhibitors reflects a generational evolution toward greater selectivity, improved tolerability, and more precise biomarker-driven use. Among CDK4/6 inhibitors, palbociclib and ribociclib are commonly considered earlier-generation orally available CDK4/6 inhibitors with predominant activity against CDK4 and CDK6, whereas abemaciclib is often regarded as a later-generation agent because of its greater CDK4 selectivity, broader continuous dosing schedule, and additional activity against other cyclin-dependent kinases. These pharmacologic differences contribute to distinct toxicity profiles, including higher rates of neutropenia with palbociclib and ribociclib and more frequent gastrointestinal toxicity with abemaciclib [2,3,4].

Similarly, PI3K pathway inhibition has evolved from less selective pan-PI3K inhibitors, which were limited by toxicity, toward isoform-selective and mutant-selective PI3Kα inhibitors. Alpelisib represents the first approved PI3Kα-selective inhibitor for PIK3CA-mutated HR+/HER2− mBC, establishing PI3Kα inhibition as a clinically actionable strategy [8]. More recently, inavolisib has emerged as a next-generation PI3Kα inhibitor with dual kinase inhibition and mutant p110α degradation, demonstrating improved efficacy and a more favorable discontinuation profile in PIK3CA-mutated endocrine-resistant disease [18,19]. Zovegalisib represents a further investigational step as an allosteric, pan-mutant-selective PI3Kα inhibitor designed to spare wild-type PI3Kα activity and potentially reduce on-target metabolic toxicity [20,21,22]. This generational progression supports the broader transition from empiric pathway inhibition toward molecularly refined, biomarker-selected treatment strategies, as shown in Table 1.

This table summarizes the main generations of CDK4/6 and PI3K inhibitors, highlighting representative agents, key pharmacologic differences, toxicity profiles, and their relevance to biomarker-guided treatment in HR+/HER2− mBC.

## 2. Methods

This narrative review synthesizes current evidence and emerging strategies for therapeutic approaches in hormone receptor-positive/human epidermal growth factor receptor 2-negative (HR+/HER2−) mBC following progression on cyclin-dependent kinase 4/6 inhibitors (CDK4/6i). This review was conducted and reported in accordance with the SANRA recommendations for narrative reviews [23]. The literature was identified through targeted searches in PubMed/MEDLINE, Embase, and the Cochrane Library from January 2010 to February 2026, using keywords such as “CDK4/6 inhibitors,” “post-CDK4/6 resistance,” “ESR1 mutations,” “PI3K/AKT inhibitors,” “oral SERDs,” “ctDNA monitoring,” and “HR+/HER2− mBC.” Additional sources included conference abstracts from ASCO, ESMO, and the San Antonio Breast Cancer Symposium, as well as ClinicalTrials.gov for ongoing trials. Two authors (HOA and NA) reviewed and selected the trials to be included in this narrative review.

Selection prioritized phase 2/3 randomized controlled trials (RCTs), real-world evidence (RWE) studies, and biomarker analyses demonstrating clinical relevance, such as pivotal trials (e.g., EMERALD, CAPItello-291, INAVO120, SERENA-6, PADA-1) and genomic profiling studies on resistance mechanisms. Inclusion focused on endocrine-based strategies, pathway inhibitors (PI3K/AKT/mTOR), and next-generation estrogen receptor degraders, excluding chemotherapy-centric or early-stage breast cancer studies. Approximately 50 main references were selected based on recency, impact factor, and alignment with review objectives, emphasizing U.S. FDA approvals as of early 2026, along with 30 additional supporting references, for a total of 80 references.

Data extraction involved summarizing study designs, patient populations, interventions, outcomes (e.g., PFS, OS, hazard ratios), resistance drivers (e.g., ESR1 mutations, PIK3CA alterations), and real-world applicability. Synthesis was thematic, organized into domains: ctDNA-guided switching, RWE performance, and molecular frameworks for co-altered disease. No formal quality assessment or meta-analysis was performed, as is typical for narrative reviews; instead, expert interpretation integrated clinical implications, limitations, and future directions. This approach allows flexibility to highlight evolving paradigms while acknowledging potential selection bias inherent to narrative formats.

Table 2 summarizes the approved biomarker-directed therapies after CDK4/6 inhibitor progression.

## 3. Genomic Profiling in Diagnostic Workup

Comprehensive genomic profiling has become an essential component of the diagnostic and therapeutic algorithm in hormone receptor-positive, HER2-negative mBC, particularly following progression on CDK4/6 inhibitors [24]. Next-generation sequencing (NGS), performed on tumor tissue and/or circulating tumor DNA (ctDNA), enables identification of actionable alterations including ESR1, PIK3CA, AKT1, and PTEN, which directly inform treatment selection [25]. In addition, genomic profiling reveals resistance mechanisms such as RB1 loss, FGFR amplifications, and cyclin pathway alterations, which may influence prognosis and guide clinical trial eligibility [5,12,26]. Importantly, ctDNA-based assays allow dynamic, minimally invasive monitoring of tumor evolution, facilitating early detection of emergent mutations—most notably ESR1—months prior to radiographic progression [9,16,21]. This enables timely therapeutic adaptation, as demonstrated in recent trials of ctDNA-guided switching strategies [15,22,27]. Given the high prevalence of polyclonal resistance, with up to one-third of tumors harboring multiple concurrent genomic drivers, repeat molecular profiling at progression is strongly recommended to refine treatment sequencing and support a precision oncology approach [5].

## 4. Real-World Evidence and Pathway-Directed Therapeutic Strategies

Unlike controlled trials, real-world cohorts include broader clinical heterogeneity, including elderly, frail, and heavily pretreated patients, making real-world evidence important for assessing treatment generalizability, subgroup benefit, and practical tolerability [28,29].

### 4.1. Alpelisib

Alpelisib, a selective PI3Kα inhibitor, represents an established biomarker-directed option for PIK3CA-mutated HR+/HER2− mBC, particularly following CDK4/6i progression. Approved by the FDA in 2019 based on the SOLAR-1 trial, where it extended median PFS to 11.0 months (vs. 5.7 months with fulvestrant alone; HR 0.65; 95% CI 0.50–0.85) in PIK3CA-mutated patients [8], alpelisib’s role in the post-CDK4/6i era has been further validated by dedicated studies and emerging RWE [30]. The phase 2 BYLieve trial specifically addressed this setting, enrolling patients post-CDK4/6i plus aromatase inhibitor, and reported a median PFS of 7.3 months with alpelisib plus fulvestrant in cohort A, with an ORR of 17% and a clinical benefit rate of 46% [30]. Recent phase 3 data from EPIK-B5 (NCT05038735) reinforced this, showing a median PFS of 7.4 months (vs. 2.8 months with fulvestrant; HR 0.52; 95% CI 0.37–0.72) in CDK4/6i-pretreated patients, highlighting consistent benefit [31].

Real-world analyses confirm alpelisib’s effectiveness beyond clinical trials, often in more heterogeneous populations. A comparative study using the Flatiron Health database matched post-CDK4/6i patients receiving alpelisib plus fulvestrant against standard therapies (e.g., everolimus plus exemestane, chemotherapy), demonstrating a median PFS of 7.3 months vs. 3.6 months (weighted HR 0.48; 95% CI 0.31–0.73), underscoring superior outcomes [32]. In a single-institution retrospective review of 27 patients (median 3 prior lines), the ORR was 12.5% with a median duration of response of 5.8 months, though treatment duration was limited by toxicity in half the cohort [33]. Broader RWE from multinational registries reports median time-to-treatment discontinuation of 4–6 months, with effectiveness maintained across subgroups including visceral disease and older patients, albeit with frequent dose adjustments [34].

Safety in real-world practice mirrors trial data, with hyperglycemia (50–60%), rash (20–30%), and gastrointestinal effects (30–40%) as the predominant AEs, often leading to dose interruptions (50–60%) or discontinuations (20–30%) [34]. Proactive management, including metformin for hyperglycemia and dermatologic prophylaxis, has improved tolerability in community settings.

### 4.2. Elacestrant

Elacestrant received FDA approval in January 2023 for ESR1-mutated HR+/HER2− mBC based on the EMERALD trial, which demonstrated median PFS of 3.8 versus 1.9 months with standard endocrine therapy (HR 0.55; *p* = 0.0005) in the ESR1-mutated population. In post hoc analysis results, patients with ≥12 months of prior CDK4/6i benefit achieved median PFS of 8.6 versus 1.9 months (HR 0.41), establishing duration of prior endocrine response as a predictive biomarker [35,36].

Multiple 2023–2026 real-world cohorts (cumulative *n*~300–750) report median time-to-next-treatment of 7.9 months overall (95% CI 7.1–9.8), frequently exceeding trial expectations. The 36subgroup analyses identify particularly favorable outcomes in earlier treatment lines (8.2–10.8 months), patients with ≥12 months prior CDK4/6i benefit (8.4 months), those without prior fulvestrant exposure (12.9 months), and individuals without prior chemotherapy (8.4 months). Patients with visceral metastases achieved 7.9 months and those with hepatic metastases achieved 7.2 months [17]. Critically, co-occurring ESR1 and PI3K pathway mutations retained clinically meaningful benefit (median time-to-next-treatment 6.3 months), supporting elacestrant as an initial endocrine-directed approach even in molecularly complex tumors [17,37]. Treatment discontinuation due to adverse events remained low, consistent with elacestrant’s manageable oral profile characterized primarily by mild-to-moderate nausea, fatigue, and arthralgia.

Table 3 presents comprehensive real-world evidence data across multiple cohorts, while Table 2 provides a comparative analysis of trial versus real-world performance metrics. While elacestrant marks a key advance in ESR1-mutated endocrine resistance, pathway-directed strategies targeting PIK3CA and AKT alterations further expand treatment options, especially post-CDK4/6i.

### 4.3. Inavolisib

Inavolisib received accelerated FDA approval in October 2024 based on the INAVO120 trial, evaluating a triplet combination of inavolisib (selective PI3Kα inhibitor with a dual mechanism of kinase inhibition and mutant p110α degradation) plus palbociclib plus fulvestrant in PIK3CA-mutated, endocrine-resistant disease. The triplet achieved median PFS of 15.0 months at primary analysis versus 7.3 months with placebo plus palbociclib plus fulvestrant (HR 0.43; *p* < 0.0001) [18]. Updated data at 34.2 months median follow-up demonstrated sustained benefit with median PFS of 17.2 months [19].

Final OS analysis revealed the first survival benefit for a PI3K pathway inhibitor: median OS 34.0 months (95% CI 29.3–39.1) versus 27.0 months (95% CI 23.1–31.9; HR 0.67; *p* = 0.019) [19]. Time to chemotherapy initiation was substantially prolonged: 35.6 versus 12.6 months (HR 0.43), representing nearly two years’ delay in cytotoxic exposure [38]. Inavolisib’s safety profile demonstrates meaningful improvements over earlier-generation PI3K inhibitors, with treatment discontinuation due to adverse events occurring in only 6.8% versus historically 25% with alpelisib [8]. Grade 3–4 hyperglycemia occurred in approximately 20% versus 36–65% with alpelisib, attributed to inavolisib’s enhanced selectivity and intermittent dosing schedule (21 days on, 7 days off) [18,19]. These data establish inavolisib as the preferred PI3K pathway inhibitor for the 30–40% of patients harboring PIK3CA mutations.

Complete INAVO120 trial design, efficacy endpoints including OS data, and comparative safety profile versus alpelisib are detailed in Table 2 and Table 4.

### 4.4. Capivasertib

Capivasertib, a pan-AKT inhibitor, received FDA approval in November 2023 based on the CAPItello-291 trial. In the overall intention-to-treat population, capivasertib plus fulvestrant achieved median PFS of 7.2 versus 3.6 months (HR 0.60; *p* < 0.001). In the biomarker-altered subgroup harboring AKT1, PIK3CA, or PTEN aberrations (41% of the trial population), median PFS was 7.3 versus 3.1 months (HR 0.50; *p* < 0.001) [39]. Approximately 70% of patients had received prior CDK4/6i exposure, validating activity in heavily pretreated populations [40,41]. Capivasertib’s biomarker-inclusive design collectively addresses 40–50% of patients—representing broader applicability than PIK3CA-selective agents. The safety profile demonstrates substantially lower rates of severe hyperglycemia compared with PI3K inhibitors (all grades hyperglycemia of 16.3% vs. ~64% with alpelisib), though characteristic toxicities include rash (21.2% grade ≥ 3) and diarrhea (9.3% grade ≥ 3) [24,42]. Emerging real-world evidence suggests modest PFS of 5–7 months in pathway-altered disease with manageable toxicity [43]. Emerging real-world findings from a large database analysis of US patients (*n* = 412 patients with MBC) demonstrate the effectiveness of capivasertib + fulvestrant in real-world practice. Clinical outcomes in second-line (2 L) and third-line (3 L) closely match those observed in the CAPItello-291 Phase 3 randomized controlled trial, which supported FDA approval of the capivasertib + fulvestrant regimen. Numerically improved outcomes were observed in patients who used capivasertib in earlier vs. later line settings (2 L and 3 L median rwTTNT were 7.1 mos (IQR 5.8, NE) and 6.9 mos (IQR 6.1, 7.8), respectively; 2 L and 3 L median rwTTD were 6.9 mos (IQR 5.3, NE) and 6.6 mos (IQR 5.2, 7.1), respectively) [44].

In exploratory ctDNA analyses from Phase 3 CAPItello-291, clinical benefit (PFS) of capivasertib + fulvestrant in the ctDNA-altered group was consistent with the primary tissue-based analysis and irrespective of ESR1m. Notably, patients receiving capivasertib + fulvestrant in 2 L post AI + CDK4/6i who had ESR1 and PIK3CA/AKT/PTEN alterations achieved a 7-month mPFS benefit, regardless of the duration of prior CDK4/6i + ET [45].

Table 2 and Table 4 present complete CAPItello-291 data including biomarker-stratified analyses and comparative positioning relative to other pathway inhibitors.

### 4.5. Everolimus

Everolimus plus exemestane was established in the pre-CDK4/6 inhibitor era by the BOLERO-2 trial, which demonstrated a median PFS of 7.8 versus 3.2 months (HR 0.45) in aromatase inhibitor-resistant HR+/HER2− mBC [38]. However, its activity appears to be attenuated in contemporary post-CDK4/6i populations. Recent real-world cohorts (2024–2025) report a median PFS of 5.3 months (95% CI 4.6–7.1) in patients previously exposed to CDK4/6 inhibitors compared with 6.7 months in CDK4/6i-naïve patients (*p* = 0.046), with corresponding median OS of 21.8 versus 27.3 months (*p* = 0.01).

In current practice, everolimus retains a role in patients without actionable ESR1 or PI3K pathway alterations and in later treatment lines following exhaustion of oral SERDs and pathway-targeted agents. Nevertheless, its clinical utility is limited by a characteristic toxicity profile (including stomatitis, fatigue, hyperglycemia, and pneumonitis), which results in treatment discontinuation in approximately 20 to 30% of patients and dose reductions in 30 to 40% [38,46]. Accordingly, everolimus is generally positioned as a later-line endocrine-based option rather than a preferred early post-CDK4/6i strategy.

Table 2 provides a systematic comparison of approved agents, summarizing regulatory status, biomarker selection, efficacy benchmarks from clinical trials and real-world studies, toxicity profiles, and evidence-based positioning. Collectively, these data support a biomarker-driven treatment framework in the post-CDK4/6i setting, integrating molecular stratification with real-world performance to guide individualized therapeutic sequencing in HR+/HER2− mBC.

## 5. Genomic Resistance Architecture and Clinical Decision Frameworks

Co-occurrence of ESR1 mutations with PI3K/AKT/PTEN pathway alterations in second-line progression affects approximately 8.2% of patients following first-line progression [25], substantially complicating therapeutic decision-making in the absence of head-to-head sequencing trials [24,47]. These genomic patterns reflect both intrinsic resistance present before therapy and acquired clonal evolution under CDK4/6i selective pressure. Clinical management therefore requires integration of molecular context with disease kinetics, prior endocrine sensitivity, presence of pathway-dominant alterations, visceral crisis, and anticipated treatment tolerability.

Comprehensive whole-exome sequencing of 59 CDK4/6i-exposed tumors identified eight distinct resistance mechanisms in 66% of cases, with approximately 29% harboring multiple concurrent drivers—highlighting substantial polyclonal complexity [5]. Identified alterations included RB1 biallelic loss, AKT1 mutations, RAS pathway activation (each ~10%), AURKA and CCNE2 amplification, ERBB2 mutations, FGFR2 alterations, and complete estrogen receptor loss. Additional studies have demonstrated acquired RB1 mutations emerging during CDK4/6i therapy (~5%) [9], FAT1 loss driving CDK6 upregulation via Hippo pathway dysregulation [42], and CCNE1/CCNE2 amplification enabling CDK2-mediated cell cycle bypass independent of CDK4/6 signaling (estimated 5–10%) [26]. FGFR amplifications, particularly FGFR1, have been detected in a substantial proportion of post-CDK4/6i tumors (15–41% across ctDNA cohorts), representing investigational but potentially actionable targets [12,48]. FGFR alterations remain early-phase targets without integrated standard-of-care options, though ongoing trials are evaluating selective FGFR inhibitors [49].

FGFR alterations, particularly FGFR1 amplification, represent an important but not yet fully actionable resistance mechanism in HR+/HER2− mBC. Although FGFR amplification has been associated with endocrine resistance, disease progression, and reduced sensitivity to CDK4/6-based strategies, no FGFR inhibitor is currently established as a standard-of-care option in this setting. Early-phase studies are evaluating FGFR inhibition as part of rational combination strategies, including endocrine therapy and CDK4/6 inhibition, in tumors with FGFR pathway activation. For example, clinical studies have explored pan-FGFR inhibitors such as erdafitinib in combination with fulvestrant and palbociclib, supporting the biologic rationale for targeting ER, CDK4/6, and FGFR signaling together in resistant HR+/HER2− disease. However, these approaches remain investigational, and further biomarker-selected trials are required to define which FGFR-altered patients derive meaningful clinical benefit [50].

Importantly, resistance alterations such as ESR1 and PIK3CA mutations function primarily as predictive biomarkers guiding targeted therapy selection, whereas other genomic features—including TP53 mutations and elevated circulating tumor DNA (ctDNA) fraction—serve predominantly prognostic roles reflecting tumor burden and genomic instability. In the BYLieve ctDNA substudy, low ctDNA fraction (<10%) at progression was associated with markedly superior PFS compared with high ctDNA burden (16.7 vs. 5.4 months; HR 0.31) [51]. Similarly, in the AURORA molecular screening program, TP53 mutations (HR 1.59) and acquired ESR1 mutations (HR 3.10) independently predicted inferior PFS [52]. These findings underscore the dual importance of identifying actionable resistance drivers and quantifying global genomic risk.

**Figure 1:**
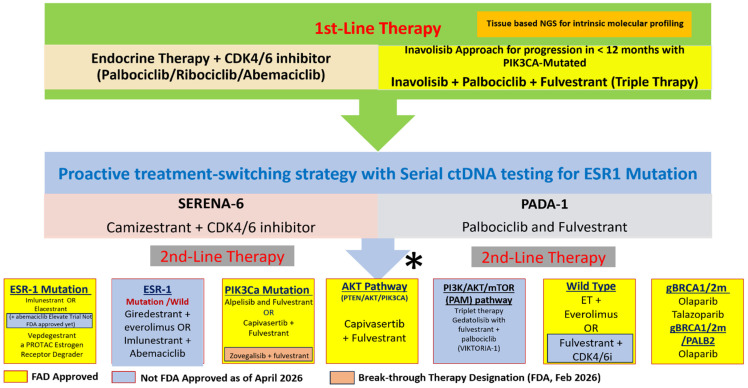
Proposed biomarker-directed treatment algorithm for HR+/HER2− mBC following CDK4/6 inhibitor progression. First-line therapy consists of endocrine therapy plus a CDK4/6 inhibitor. Comprehensive molecular profiling using tissue-based NGS and/or circulating tumor DNA (ctDNA) is recommended at baseline and at progression to guide treatment selection according to dominant molecular alterations, including ESR1, PIK3CA, AKT/PTEN/PIK3CA pathway alterations, PI3K/AKT/mTOR pathway activation, gBRCA1/2/PALB2, and wild-type disease. Yellow boxes indicate FDA-approved treatment options, purple boxes indicate investigational or not-yet-FDA-approved approaches as of April 2026, and the orange box indicates agents or combinations with FDA breakthrough therapy designation. In patients with PIK3CA-mutated tumors and early progression, including recurrence while on or within 12 months of completing adjuvant endocrine therapy, inavolisib-based triplet therapy represents an evidence-supported strategy. The middle section highlights proactive serial ctDNA monitoring for emergent ESR1 mutations, enabling early treatment switching before radiographic progression, as evaluated in SERENA-6 with camizestrant plus a CDK4/6 inhibitor and PADA-1 with fulvestrant plus palbociclib. Investigational strategies are indicated where applicable. * In patients with visceral crisis, rapid symptomatic progression, endocrine-refractory disease, or exhaustion of biomarker-directed endocrine-based options, transition to single-agent chemotherapy or ADC therapy should be considered, with trastuzumab deruxtecan favored for HER2-low/HER2-ultralow disease and sacituzumab govitecan considered in later-line HER2-negative disease.

From a clinical implementation perspective, these data strongly support repeat NGS—preferably incorporating ctDNA analysis—at radiologic progression. Molecular tumor board review should prioritize dominant resistance drivers while acknowledging polyclonal architecture that may limit single-pathway strategies. In selected cases, combination or sequential multi-pathway approaches may be more appropriate than isolated targeted therapy. Table 5 synthesizes key genomic resistance studies, including whole-exome sequencing analyses, serial ctDNA investigations, and large-scale molecular screening programs that collectively define the contemporary resistance landscape. Figure 1 summarizes the proposed biomarker-directed sequencing framework.

### 5.1. Decision Framework for Co-Altered Populations

In patients demonstrating endocrine-sensitive disease characteristics—defined by ≥12 months of clinical benefit on the most recent prior endocrine therapy plus CDK4/6i regimen, absence of visceral crisis presentations, and manageable disease kinetics—elacestrant monotherapy represents the preferred initial strategy. Real-world evidence supports durable benefits even in co-mutated ESR1 and PI3K pathway-altered tumors (median time-to-next-treatment 6.3 months), comparable to pathway wild-type outcomes [17]. This approach capitalizes on elacestrant’s excellent tolerability profile, oral administration, and broad activity across ESR1 mutation variants, reserving pathway-directed therapies for subsequent lines following elacestrant progression. Conversely, patients exhibiting pathway-dominant features—including specific high-impact hotspot mutations (PIK3CA H1047R, E545K), rapid disease progression kinetics, liver-predominant metastatic burden, or acceptable hyperglycemia tolerance—may derive superior benefit from upfront pathway targeting with inavolisib triplet therapy (in PIK3CA-mutated disease) or capivasertib plus fulvestrant (in broader AKT/PIK3CA/PTEN-altered populations). CAPItello-291 demonstrated particularly robust efficacy in pathway-altered subgroups (median PFS 7.3 vs. 3.1 months; HR 0.50) [39]. An illustrative clinical case exemplifies this framework: a patient harboring co-occurring ESR1 Y537S and PIK3CA E545K mutations following 18 months of palbociclib plus letrozole benefit would typically receive elacestrant initially, with anticipated time-to-next-treatment of 6–8 months, reserving inavolisib or capivasertib for subsequent progression. If rapid progression occurs on elacestrant (<4–6 months), switching to pathway inhibition addresses pathway-dominant resistance.

The clinical decision algorithm for co-altered tumor populations (Figure 1) integrates biomarker-driven sequencing principles, prioritizing oral selective estrogen receptor degraders (elacestrant) in ESR1-mutated, endocrine-sensitive cases while directing PI3K/AKT pathway-altered tumors toward capivasertib plus endocrine therapy based on pathway dominance features. This framework synthesizes evidence from Table 1, Table 2, Table 3, Table 4, Table 5, Table 6 and Table 7, operationalizing molecular profiling results into evidence-based treatment selection.

### 5.2. Therapeutic Strategies for Patients Lacking Actionable Genomic Alterations in Post-CDK4/6i HR+/HER2− mBC

In patients without actionable ESR1, PI3K/AKT/PTEN, germline BRCA1/2, or other targetable alterations, the decision framework prioritizes maintaining endocrine-based therapy when feasible, particularly in those with indolent disease or low burden. Everolimus combined with fulvestrant or exemestane represents a standard option, supported by BOLERO-2 data showing improved PFS (7.8 months vs. 3.2 months with exemestane alone; HR 0.45) in endocrine-resistant settings [53], though real-world efficacy post-CDK4/6i is modest (mPFS ~4–5 months) [46]. For select patients with prolonged prior CDK4/6i benefit (>12 months), continuing CDK4/6 inhibition (e.g., abemaciclib or ribociclib) with an endocrine switch may extend control, as suggested by MAINTAIN and postMONARCH trials [54,55].

In cases of rapid progression, visceral crisis, or endocrine resistance, transition to chemotherapy (e.g., capecitabine, taxanes, or eribulin) or antibody-drug conjugates is advised. Sacituzumab govitecan offers superior OS (HR 0.79 vs. chemotherapy) in later lines, while trastuzumab deruxtecan is preferred for HER2-low tumors [56].

Accordingly, chemotherapy and antibody–drug conjugates should be positioned as essential components of the treatment algorithm once endocrine-based and biomarker-directed options are exhausted, or earlier in patients with visceral crisis, rapidly progressive disease, symptomatic organ compromise, or clear endocrine-refractory biology. Single-agent chemotherapy remains appropriate when rapid cytoreduction is needed or when targeted options are unavailable, whereas ADCs increasingly provide preferred later-line options because they combine cytotoxic delivery with biomarker-defined or antigen-directed targeting. In HR+/HER2− mBC, trastuzumab deruxtecan is particularly relevant for HER2-low or HER2-ultralow disease, while sacituzumab govitecan is an important option after prior endocrine therapy and chemotherapy exposure. Integrating ADC eligibility into the post-CDK4/6 algorithm ensures that patients are not kept on ineffective endocrine-based therapy when the disease biology has shifted toward endocrine resistance [56].

## 6. Emerging Molecularly Targeted Therapies

Vepdegestrant (ARV-471), a proteolysis-targeting chimera (PROTAC) estrogen receptor degrader, represents the first phase 3 validation of targeted ER degradation technology in solid tumors [57,58]. In the VERITAC-2 trial, patients with ESR1-mutated HR+/HER2− advanced breast cancer progressing on endocrine therapy plus CDK4/6 inhibition achieved a median PFS of 5.0 months (95% CI 3.7–7.4) with vepdegestrant compared with 2.1 months (95% CI 1.9–3.5) with fulvestrant (HR 0.57–0.58; *p* < 0.001), confirming activity in molecularly selected disease (Table 4). Treatment discontinuation due to adverse events occurred in 2.9%, with fatigue and nausea as the predominant toxicities. Regulatory review is ongoing [59,60,61].

Imlunestrant demonstrated clinically meaningful efficacy in the phase 3 EMBER-3 trial [60]. In ESR1-mutated tumors, monotherapy achieved a median PFS of 5.5 versus 3.8 months (HR 0.62; *p* = 0.0007), with an OS signal favoring treatment (34.5 vs. 23.1 months; HR 0.60; *p* = 0.0043) (Table 4) [61,62,63]. Notably, combination therapy with abemaciclib—representing continued CDK4/6 inhibition beyond prior exposure—extended median PFS to 9.4 months compared with 5.5 months for monotherapy (HR 0.57; *p* < 0.001), demonstrating activity irrespective of ESR1 status and supporting the concept of rational CDK4/6 re-challenge in selected patients (Table 4) [61].

Giredestrant, evaluated in phase 3 persevERA [49], did not meet its primary PFS endpoint in the overall post-CDK4/6 population (Table 6) [49,64,65,66]. Although a numerical improvement was observed in the ESR1-mutated subgroup, this did not reach statistical significance. These findings underscore both the biological promise and clinical challenges of oral SERDs in heavily pretreated disease [11,66,67].

**Table 6:** Emerging ER degraders and rational combination strategies.

Trial (NCT)	Phase	Therapeutic Class	Population	Intervention	Primary Endpoint	Key Findings	Status
VERITAC-2 (NCT05654623)	3	PROTAC ER degrader	ESR1-mut HR+/HER2− post-ET + CDK4/6i	Vepdegestrant vs. fulvestrant	PFS (ESR1-mut)	5.0 vs. 2.1 mo; HR 0.57–0.58; low AE discontinuation	Regulatory review ongoing
EMBER-3 (NCT04975308)	3	Oral SERD ± CDK4/6i	HR+/HER2− advanced BC	Imlunestrant vs. SOC; Imlunestrant + abemaciclib	PFS	ESR1-mut: 5.5 vs. 3.8 mo (HR 0.62); Combo: 9.4 vs. 5.5 mo (HR 0.57)	Regulatory status evolving
PERSEVERE (NCT04546009)	3	Oral SERD	HR+/HER2− post-CDK4/6i	Giredestrant vs. physician’s choice ET	PFS	Primary endpoint not met; numerical ESR1-mut benefit	Development strategy ongoing
ELEVATE	2	SERD-based combinations	HR+/HER2− post-CDK4/6i	Elacestrant + everolimus/abemaciclib	PFS	8.3 mo (everolimus); 14.3 mo (abemaciclib)	Proof-of-concept
ADELA (NCT06382948)	3	SERD + mTOR inhibitor	ESR1-mut HR+/HER2−	Elacestrant + everolimus vs. monotherapy	PFS	Ongoing	Recruiting
INAVO121	3	PI3K inhibitor comparison	PIK3CA-mut HR+/HER2−	Inavolisib vs. alpelisib	PFS	Direct comparative efficacy study	Active enrollment
ReDiscover (NCT05216432) ReDiscover-2 (NCT06982521)	1/2 (Ph 3 ongoing)	PIK3CA-mutated HR+/HER2− mBC post-CDK4/6i	Zovegalisib + fulvestrant (Ph 3: vs. capivasertib + fulvestrant)	Zovegalisib + fulvestrant (Ph 3: vs. capivasertib + fulvestrant)	ReDiscover = Safety, ReDiscover-2 = PFS	Median PFS 11.1 mo (95% CI 7.3–13.0) at recommended Phase 3 dose 400 mg BID with food	Breakthrough Therapy Designation (FDA, Feb 2026); Phase 3 ongoing

Gedatolisib, a highly potent multitarget inhibitor of the PI3K/AKT/mTOR (PAM) signaling pathway, has demonstrated significant clinical activity in the phase 3 VIKTORIA-1 trial, validating PAM pathway inhibition as a therapeutic strategy in hormone receptor-positive, HER2-negative advanced breast cancer. In this study, patients with disease progression after endocrine therapy plus CDK4/6 inhibition received gedatolisib with fulvestrant with or without palbociclib versus fulvestrant alone. In patients with PIK3CA-wild-type tumors, median PFS was 9.3 months with the gedatolisib triplet (HR 0.24) and 7.4 months with the gedatolisib doublet (HR 0.33), compared with 2.0 months with fulvestrant alone, with consistent benefit regardless of the prior CDK4/6 inhibitor used, including in patients who experienced progression on first-line therapy within less than 6 months [68,69].

Treatment discontinuation due to treatment-related adverse events occurred in 2.3% and 3.1% of patients in the triplet and doublet arms, respectively, indicating good tolerability [68]. These results confirm the PAM pathway as a key molecular driver in PIK3CA-wild-type disease, and gedatolisib plus fulvestrant, with or without palbociclib, may represent a new standard of care after CDK4/6 inhibitor progression.

Combination strategies are increasingly being explored to enhance endocrine backbone efficacy and delay resistance. The results from the phase 2 ELEVATE trial, an open-label, umbrella study [69] reported median PFS of 8.3 months with elacestrant plus everolimus and 14.3 months with elacestrant plus abemaciclib in the post-CDK4/6 setting (Table 4) [17,28]. The ongoing phase 3 ADELA trial is evaluating elacestrant plus everolimus versus elacestrant monotherapy in ESR1-mutated disease and may clarify the role of oral SERD doublet combinations [70] (Table 6). Additionally, INAVO121 is directly comparing inavolisib with alpelisib in PIK3CA-mutated tumors, addressing comparative pathway inhibition strategies [71] (Table 6). The CAPItello-292 Phase III study is evaluating capivasertib + CDK4/6i + fulvestrant in 1L patients with and without PIK3CA/AKT1/PTEN alterations, and the Victoria-2 Phase III trial evaluates gedatolisib + CDK4/6 + fulvestrant in 1L patients with HR+/HER2− advanced breast cancer (ABC) who are endocrine therapy resistant [67].

Zovegalisib (RLY-2608) plus fulvestrant represents a next-generation advancement in PI3K pathway inhibition. The U.S. Food and Drug Administration granted Breakthrough Therapy designation in February 2026 to zovegalisib (an allosteric, pan-mutant-selective PI3Kα inhibitor) in combination with fulvestrant for adults with PIK3CA-mutated, hormone receptor-positive, HER2-negative locally advanced or mBC following progression on or after a CDK4/6 inhibitor [20]. This designation, supported by data from the phase 1/2 ReDiscover trial, underscores the evolving role of precision PI3Kα inhibition in endocrine-resistant HR+/HER2− disease. In heavily pretreated post-CDK4/6i patients receiving the recommended phase 3 dose (400 mg BID with food) plus fulvestrant, the regimen achieved a median PFS of 11.1 months (95% CI: 7.3–13.0), with highly consistent benefit across kinase-domain and non-kinase-domain PIK3CA mutations and encouraging objective response rates (43% overall; 52% in second-line) [21]. The safety profile was notable for markedly reduced wild-type-sparing effects, resulting in predominantly low-grade, manageable hyperglycemia and limited treatment discontinuations. A global phase 3 trial (ReDiscover-2) is actively enrolling and directly compares zovegalisib plus fulvestrant versus capivasertib plus fulvestrant in this exact setting, offering the potential to further refine second-line options for the approximately 30–40% of patients with PIK3CA-mutated tumors [20,22].

Collectively, next-generation ER degraders and rational combination regimens aim to achieve deeper estrogen receptor suppression and multi-pathway blockade. Their mechanistic rationale is supported by prior evidence from EMERALD (elacestrant), CAPItello-291 (capivasertib) [72], and INAVO120 (inavolisib) [30], which demonstrated that molecularly selected strategies improve outcomes when matched to dominant resistance biology (Table 4). Together, these data reinforce the importance of comprehensive genomic profiling and dynamic resistance monitoring to guide therapy selection following CDK4/6 inhibitor progression.

Table 5 and Table 6 illustrate this heterogeneity through systematic compilation of variable inclusion criteria, biomarker selection methodologies, and endpoint definitions across pivotal trials.

## 7. Circulating Tumor DNA-Guided Proactive Switching for ESR1 Mutations: Evidence and Implementation

Traditional therapeutic paradigms have relied upon radiographic evidence of disease progression per RECIST criteria to trigger treatment modifications, an approach that permits continued tumor expansion and progressive accumulation of resistance mechanisms under ongoing selective pressure. Circulating tumor DNA enables minimally invasive, real-time molecular surveillance, detecting emergent resistance alterations—particularly ESR1 mutations—months prior to radiographic manifestations [15,27]. Two landmark phase 3 trials have established that proactive, ctDNA-guided therapeutic switching meaningfully extends PFS and quality of life.

### 7.1. SERENA-6 Trial

The SERENA-6 trial (NCT04964934) prospectively enrolled patients with ER+/HER2− advanced breast cancer receiving first-line aromatase inhibitor plus CDK4/6i for at least 6 months, implementing serial ctDNA monitoring via Guardant360 CDx every 2–3 months. Among 3256 patients screened, 315 developed emergent ESR1 mutations without radiographic progression (Among 3325 patients screened, 3256 underwent serial ctDNA testing; 548 (16.8%) developed emergent ESR1 mutations, of whom the first 315 were randomized 1:1 to camizestrant + CDK4/6i [15,27]. A crude estimate of the proportion of patients with emergent ESR1m during the study period is 42%, calculated from the 548 patients with a positive test/(the number of patients tested for ESR1m [*n* = 3256] minus the number of patients that were still ongoing in surveillance when screening closed [*n* = 1949]). These patients did not undergo further ESR1m testing and could have had ESR1m arising later on. Then, 315 patients were randomized 1:1 to switch to camizestrant (next-generation oral SERD) while continuing CDK4/6i or maintaining aromatase inhibitor plus CDK4/6i [27,73,74].

At median follow-up of 12.6 months, median PFS was 16.0 months (95% CI 12.7–18.2) with camizestrant versus 9.2 months (95% CI 7.2–9.5) with continued aromatase inhibitor (HR 0.44; 95% CI 0.31–0.60; *p* < 0.0001), representing a 56% risk reduction [27]. One-year PFS rates were 60.7% versus 33.4%. Benefit was consistent across CDK4/6i type, age, visceral disease status, and ESR1 variant. Median time to deterioration in global health status was 21.0 versus 6.4 months (HR 0.54; *p* = 0.001) [73,74]. Treatment discontinuation due to adverse events remained low (1.3% vs. 1.9%). Molecular analyses demonstrated a 100% median reduction in ESR1 mutant allele frequency at 8 weeks with camizestrant versus a 66.7% increase with continued aromatase inhibitor, validating the biological rationale for early intervention [16,74,75]. Table 7 summarizes complete trial design, population characteristics, primary endpoints, and key efficacy outcomes for SERENA-6 and PADA-1 ctDNA-guided switching trials.

**Table 7:** ctDNA-guided switching trials.

Trial	Strategy	Population	Median PFS (mo)	HR	Key Finding
SERENA-6	Camizestrant + CDK4/6i vs. AI + CDK4/6i	Emergent ESR1	16.0 vs. 9.2	0.44	Proactive switch prolongs PFS
PADA-1	Fulvestrant + palbo vs. AI + palbo	Rising ESR1	11.9 vs. 5.7	0.61	Early intervention beneficial

### 7.2. PADA-1 Trial

The PADA-1 trial provided complementary validation through a distinct design, randomizing patients with rising ESR1 mutations (detected via droplet digital PCR every 2 months during palbociclib plus aromatase inhibitor) to switch to fulvestrant plus palbociclib or continue aromatase inhibitor plus palbociclib. Among 1017 patients screened, 172 (16.9%) developed ESR1 mutations prior to conventional progression [16]. Fulvestrant switching doubled median PFS: 11.9 months (95% CI 9.4–13.5) versus 5.7 months (95% CI 3.9–7.5; HR 0.61; *p* = 0.0040) [73]. Median time to ESR1 detection was 18 months, providing substantial lead time for intervention. Molecular characterization revealed polyclonal ESR1 mutations increased from 26.3% at initial detection to 69.2% at eventual progression, underscoring rapid clonal diversification and narrowing therapeutic windows [16].

### 7.3. Implementation Considerations

These trials collectively examined serial ctDNA monitoring during first-line CDK4/6i plus endocrine therapy, with active switching upon ESR1 emergence, which extends endocrine-based treatment duration by 5–7 months. Optimal implementation requires monitoring every 2–3 months during stable disease, utilizing platforms with analytical sensitivity < 0.1–0.5% variant allele fraction. Both Guardant360 CDx and targeted droplet digital PCR demonstrate clinical validity, though comprehensive NGS panels offer additional detection of concurrent pathway alterations (PIK3CA, AKT1, PTEN) [75,76]. Turnaround times of 7–14 days are generally acceptable for asymptomatic patients. False-negative results occur more frequently in low-shedding disease (bone-only metastases), necessitating integration with conventional imaging surveillance. Clinical implementation requires integration with imaging surveillance, particularly in low-shedding disease such as bone-only metastases. Cost-effectiveness modeling suggests potential economic favorability when considering prolonged PFS, delayed transition to expensive therapies, and maintained quality of life, though definitive health economic evaluations across diverse healthcare systems remain needed [14]. As of February 2026, ctDNA-guided switching has not yet been incorporated into major international guidelines but is positioned to influence imminent updates given the strength of phase 3 evidence [64,77,78,79]. Together, these trials provide compelling evidence to reconsider current monitoring guidelines and pave the way for integrating molecular response surveillance as a standard component of care in HR+/HER2− mBC. Beyond molecularly guided switching strategies, the therapeutic landscape has further evolved through pathway-directed agents that target dominant resistance biology following CDK4/6 inhibitor progression.

## 8. Limitations

This narrative review must be interpreted in light of several methodological and translational limitations. First, the evidence base comprises heterogeneous clinical trials that vary considerably in design, patient selection criteria, biomarker stratification methods, control arms, and endpoint definitions. These differences preclude direct cross-trial comparisons and limit the feasibility of formal meta-analysis. Nonetheless, synthesizing this dataset was essential to inform real-world clinical decision-making in a landscape where head-to-head sequencing trials remain absent. Second, the absence of prospective randomized trials comparing post-CDK4/6i treatment sequences in molecularly defined subgroups (e.g., ESR1-mutated vs. PI3K-altered tumors) necessitates reliance on indirect comparisons and expert consensus rather than Level 1 evidence. The treatment decision framework developed in this review is thus proposed as a provisional model grounded in available clinical, molecular, and real-world data.

Third, several of the key insights regarding agent efficacy, particularly for elacestrant and pathway-directed therapies, derive from real-world evidence (RWE). While RWE enhances external validity, it is subject to well-documented limitations including retrospective data collection, non-standardized biomarker testing, selection bias, and variability in outcome reporting. Fourth, long-term outcome data—especially OS and quality-of-life metrics—remain immature for many recently approved agents, such as imlunestrant and inavolisib. Nevertheless, their inclusion is justified based on their regulatory approval, promising surrogate endpoints, and substantial clinical relevance in current practice. Fifth, this review was deliberately scoped to focus on oral selective estrogen receptor degraders (SERDs), PI3K/AKT/mTOR pathway inhibitors, and PROTAC degraders. Cytotoxic chemotherapy, antibody-drug conjugates, and emerging immunotherapeutic options were not included, and therefore the review does not provide exhaustive coverage of the entire post-CDK4/6i treatment landscape. Finally, cost-effectiveness data remain limited for most novel endocrine-based therapies, and disparities in access across health systems globally may constrain the implementation of biomarker-driven paradigms. Future studies should prioritize comprehensive economic evaluations and implementation science approaches to support equitable adoption. Importantly, each analysis included in this review—ranging from genomic resistance mapping and ctDNA monitoring paradigms to treatment decision algorithms and comparative trial synthesis—was selected to directly address the review’s central aim: to operationalize biomarker-informed therapeutic sequencing strategies in HR+/HER2− mBC following CDK4/6 inhibitor progression. These analytical choices reflect clinical practice gaps and align with the translational urgency to refine treatment personalization amid increasing molecular complexity.

## 9. Conclusions and Future Directions

The therapeutic landscape following CDK4/6 inhibitor progression in HR+/HER2− mBC has evolved substantially, driven by biomarker-directed therapies and improved understanding of resistance biology. Recent trials, including SERENA-6 and PADA-1, demonstrate that serial circulating tumor DNA (ctDNA) monitoring with preemptive therapeutic modification upon emergent ESR1 mutation detection can extend PFS by approximately 5–7 months and delay quality-of-life deterioration. Although these data support a shift toward earlier molecularly informed adaptation, ctDNA-guided proactive switching remains investigational and has not yet been incorporated into major international guidelines.

Looking forward, ctDNA is expected to evolve from a resistance-detection tool into a dynamic treatment-decision platform in HR+/HER2− mBC. Beyond identifying emergent ESR1 mutations, serial ctDNA assessment may help define molecular progression before radiographic progression, quantify tumor burden through variant allele fraction dynamics, identify co-emerging resistance pathways such as PIK3CA, AKT1, PTEN, RB1, and FGFR alterations, and guide earlier transition to matched targeted therapies or rational combination strategies [5,15,16,27,51,75,76]. Future clinical trials should evaluate ctDNA-guided escalation, de-escalation, and sequencing approaches across multiple resistance mechanisms, rather than focusing on single-gene alterations alone [15,16,73,74]. Integration of ctDNA kinetics with imaging, clinical disease tempo, and artificial intelligence-based predictive models may further personalize treatment selection, delay ineffective therapy, reduce unnecessary toxicity, and optimize the timing of chemotherapy or antibody–drug conjugates [27,51,75,76]. However, broad implementation will require assay standardization, clinically validated thresholds for actionability, prospective evidence of survival and quality-of-life benefit, cost-effectiveness data, and equitable access across healthcare systems [75,76,77,78,79].

In routine practice, comprehensive NGS testing at radiologic progression has become central to therapeutic individualization. Molecular profiling identifies actionable alterations—including ESR1, PIK3CA, AKT1, PTEN, and FGFR—as well as prognostic markers such as TP53 mutations and elevated ctDNA fractions. Integrating these findings with clinical parameters, particularly duration of endocrine sensitivity and disease kinetics, enables rational sequencing decisions that move beyond empiric approaches.

Endocrine-directed therapy continues to provide meaningful benefits in selected patients. Real-world data support the durability of elacestrant in ESR1-mutated, endocrine-sensitive disease. Pathway-directed strategies have further expanded options: inavolisib has demonstrated the first OS advantage for a PI3K pathway inhibitor in PIK3CA-mutated disease, and capivasertib provides activity across broader AKT/PIK3CA/PTEN-altered populations. These advances underscore the importance of aligning treatment selection with dominant resistance pathways.

Emerging evidence suggests that combination strategies may be more effective than single-agent approaches in overcoming resistance. Triplet regimens incorporating pathway inhibition, optimized endocrine partners, and continued CDK4/6 blockade exemplify this strategy. Notably, CDK4/6 inhibition beyond prior exposure has shown clinical benefit in selected patients, particularly when combined with alternative endocrine backbones, indicating that resistance to one CDK4/6-based regimen does not universally preclude subsequent benefit.

Overall, post-CDK4/6 inhibitor management is moving from empiric endocrine sequencing toward biomarker-informed precision care. NGS and ctDNA profiling enable detection of actionable resistance mechanisms, while emerging data support rational combinations and proactive molecular surveillance in selected patients. Future priorities include prospective sequencing trials, validation of ctDNA-guided strategies beyond ESR1 mutations, optimization of combination regimens, and cost-effectiveness studies to support equitable implementation.

## Figures and Tables

**Table 1:** Generational evolution of CDK4/6 and PI3K inhibitors.

Therapeutic Class	Generation/Category	Examples	Key Features
CDK4/6 inhibitors	Earlier-generation CDK4/6 inhibitors	Palbociclib, ribociclib	Established first-line backbone; intermittent dosing; neutropenia common
CDK4/6 inhibitors	Later-generation/more CDK4-selective inhibitor	Abemaciclib	Greater CDK4 selectivity; continuous dosing; more gastrointestinal toxicity
PI3K inhibitors	Earlier pan-PI3K inhibitors	Buparlisib, pictilisib	Broader PI3K blockade; limited clinical use due to toxicity
PI3K inhibitors	PI3Kα-selective inhibitor	Alpelisib	Approved for PIK3CA-mutated HR+/HER2− metastatic breast cancer; hyperglycemia and rash common
PI3K inhibitors	Next-generation PI3Kα inhibitor	Inavolisib	PI3Kα inhibition plus mutant p110α degradation; improved efficacy and tolerability profile
PI3K inhibitors	Mutant-selective PI3Kα inhibitor	Zovegalisib	FDA Approved Feb 2026; designed to target mutant PI3Kα while sparing wild-type PI3Kα

**Table 2:** Approved and emerging biomarker-directed therapies after CDK4/6 inhibitor progression in HR+/HER2− mBC.

Therapeutic Class	Agent	Molecular Selection	Pivotal Trial(s)	Median PFS (Months)	OS Signal	Regulatory Status (as of Early 2026)	Clinical Positioning/Notes
Oral SERD	Elacestrant	ESR1-mutated	EMERALD	3.8 (overall); 8.6 (≥12 mo prior CDK4/6i)	No statistically significant OS benefit yet	FDA approved (2023)	Preferred in ESR1-mut endocrine-sensitive disease; best in longer prior CDK4/6i exposure
AKT inhibitor	Capivasertib	AKT1/PIK3CA/PTEN altered	CAPItello-291	7.2 (overall); 7.3 (altered)	Not mature/no clear OS benefit yet	FDA approved	Pathway-altered (AKT/PI3K/PTEN) dominant tumors; post-CDK4/6i option
PI3Kα inhibitor (triplet)	Inavolisib (triplet: +palbociclib +fulvestrant)	PIK3CA-mutated	INAVO120	15.0 months at primary analysis; 17.2 months at updated follow up	Yes (HR 0.67; mature OS benefit)	FDA approved (2024)	PIK3CA-mut endocrine-resistant tumors; triplet strategy with clear OS benefit
mTOR inhibitor	Everolimus (+exemestane)	None required	BOLERO-2	7.8 (pre-CDK4/6i era); ~3.8–5.4 in RWE post-CDK4/6i	No OS benefit	Approved (earlier line)	Later-line endocrine-based strategy; reduced efficacy post-CDK4/6i in real-world data
PI3K inhibitor	Alpelisib (+fulvestrant)	PIK3CA-mutated	SOLAR-1	11.0	No OS (numeric 7.9 mo improvement, not stat sig)	Approved	Earlier PI3K option post-CDK4/6i in PIK3CA-mut; established but hyperglycemia common
Endocrine therapy (backbone)	Fulvestrant	ER+	Various (e.g., PALOMA-3 reference)	Limited monotherapy activity post-CDK4/6i	N/A	Approved	Backbone; limited single-agent efficacy post-CDK4/6i; used in combinations
PARP inhibitor	Olaparib	Germline BRCA1/2 mutated	OlympiAD/others	N/A (PARP context)	OS benefit in gBRCA population	Approved	Germline BRCA-mutated population; post-CDK4/6i if applicable
PARP inhibitor	Talazoparib	Germline BRCA1/2 mutated/PALB2 mutated	EMBRACA	N/A (PARP context)	OS benefit in gBRCA population	Approved	Germline BRCA-mutated population; post-CDK4/6i if applicable
Oral SERD	Camizestrant	ESR1-mutated	SERENA-6 (proactive switch)	N/A (investigational)	N/A	Phase III/investigational	Investigational; proactive ctDNA-guided in ESR1-mut
Oral SERD/PROTAC ER degrader	Imlunestrant	ESR1-mutated	EMBER-3	N/A (investigational)	N/A	Phase III/region-dependent	Investigational; emerging oral SERD
PROTAC ER degrader	Vepdegestrant	ESR1-mutated	VERITAC-2	N/A (investigational)	N/A	Regulatory review/emerging	Emerging; PROTAC-based for ESR1-mut
Oral SERD	Giredestrant	ESR1-mutated	persevERA (negative primary)	N/A (investigational)	N/A	Phase III (primary endpoint negative)	Investigational; limited promise based on trial results
PI3K/mTOR inhibitor	Gedatolisib	PIK3CA-WT/PI3K pathway	VIKTORIA-1	N/A (investigational)	N/A	Phase III	Investigational; for PIK3CA wild-type pathway-driven tumors
Pan-mutant selective PI3K inhibitor	Zovegalisib (+fulvestrant)	PIK3CA-mutated	ReDiscover (Ph 1/2) ReDiscover-2 (Ph3 ongoing)	11.1	Not mature	Breakthrough Therapy Designation (FDA, Feb 2026); Ph 3 ongoing	Mutant-selective (allosteric, pan-mutant) PI3Kα inhibitor.

**Table 3:** Key real-world evidence studies post-CDK4/6 inhibitor.

Study/Agent	Study Type	Population (n)	Key Subgroups	Median TTNT/PFS (95% CI)	Median OS (95% CI)	Key Findings
Elacestrant RWE (2023–2026 cohorts)	Multicenter retrospective	ESR1-mutated HR+/HER2− mBC (n~300–750)	Overall 1–2 prior ET lines No prior fulvestrant ≥12 mo prior CDK4/6i ESR1 + PIK3CA co-mut	Overall: 7.9 mo (7.1–9.8) 1–2 prior: 8.2–10.8 mo No fulv: 12.9 mo ≥12 mo: 8.4 mo Co-mut: 6.3 mo	Not reported	RWE exceeds trial PFS Retained benefit in co-mutated Low discontinuation
Everolimus + ET Post-CDK4/6i RWE	Multicenter retrospective	HR+/HER2− mBC with prior CDK4/6i (n~200–400)	Post-CDK4/6i CDK4/6i-naïve	Post-CDK4/6i: 5.3 mo (4.6–7.1) Naïve: 6.7 mo (5.8–7.6) p = 0.046	Post-CDK4/6i: 21.8 mo (18.5–25.5) Naïve: 27.3 mo (23.2–30.2) p = 0.01	Attenuated vs. pre-CDK4/6i era Toxicity limits use (20–30% D/C)
Capivasertib RWE (emerging 2025)	Early real-world cohorts	Pathway-altered HR+/HER2− mBC post-CDK4/6i	AKT/PIK3CA/PTEN-altered	5–7 mo (limited data)	Not reported	Modest benefit Manageable toxicity with dose modifications

**Table 4:** Approved biomarker-directed therapies after CDK4/6 inhibitor progression: comparative efficacy and positioning.

Trial (NCT)	Phase	Molecular Selection	Intervention vs. Control	Primary Endpoint	Key Results	HR (95% CI)	Regulatory Status
EMERALD (NCT03778931)	3	ESR1-mutated ER+/HER2− mBC post-ET	Elacestrant vs. SOC ET	PFS (ESR1-mut)	Median PFS 3.8 vs. 1.9 mo; ≥12 mo prior ET + CDK4/6i: 8.6 vs. 1.9 mo	0.55 (0.39–0.77) (Exploratory)	FDA approved (Jan 2023)
INAVO120 (NCT04191499)	3	PIK3CA-mutated HR+/HER2− LA/mBC	Inavolisib + palbociclib + fulvestrant vs. placebo + palbociclib + fulvestrant	PFS	Median PFS 15.0 vs. 7.3 months at primary analysis; updated median PFS 17.2 vs. 7.3 months; and final OS 34.0 vs. 27.0 months; delayed chemotherapy	PFS: 0.43 (0.32–0.59); OS: 0.67 (0.49–0.91)	FDA approved (Oct 2024)
CAPItello-291 (NCT04305496)	3	HR+/HER2− advanced BC; AKT pathway-altered subgroup	Capivasertib + fulvestrant vs. placebo + fulvestrant	PFS	Overall: 7.2 vs. 3.6 mo; Altered: 7.3 vs. 3.1 mo	Overall: 0.60 (0.51–0.71); Altered: 0.50 (0.38–0.65)	FDA approved (Nov 2023)
BOLERO-2	3	AI-resistant HR+/HER2− BC (pre-CDK4/6 era)	Everolimus + exemestane vs. placebo + exemestane	PFS	7.8 vs. 3.2 mo; post-CDK4/6 RWE ~5.3 mo	0.45 (0.38–0.54)	Established therapy

**Table 5:** Key genomic profiling and resistance mechanism studies.

Study	Study Type	Population (*n*)	Key Findings	Resistance Mechanisms Identified	Clinical Implications
Wander et al. (Cancer Discovery 2020) [5]	Whole-exome sequencing	CDK4/6i-exposed tumors (*n* = 59)	8 distinct resistance mechanisms in 66% of cases 29.3% harbor ≥ 2 concurrent drivers (polyclonal)	RB1 loss (9.8%) AKT1 mutations (9.8%) RAS pathway (9.8%) AURKA amp CCNE2 amp ERBB2 mutations FGFR2 alterations ER loss	Multi-targeted strategies needed Single-pathway approaches inadequate in 30% due to polyclonal resistance
PALOMA-3 ctDNA Analysis	Serial liquid biopsy	Palbociclib-treated patients	Acquired RB1 mutations during treatment ESR1 mutations emerge on therapy	RB1 mutations (~5%) ESR1 mutations (increasing VAF)	Direct CDK4/6i target inactivation Serial monitoring detects resistance early
BYLieve ctDNA Substudy	Prospective ctDNA profiling	PIK3CA-mutated mBC post-CDK4/6i	ctDNA fraction strongly prognostic Low ctDNA (<10%) predicts superior PFS	High ctDNA burden associated with worse outcomes	Low ctDNA: PFS 16.7 mo High ctDNA: PFS 5.4 mo HR 0.31 ctDNA quantification is prognostic biomarker
AURORA Molecular Screening Program	Large-scale genomic profiling	HR+/HER2− mBC	TP53 and ESR1 mutations independently predict worse outcomes	TP53 mutations: HR 1.59 for PFS ESR1 mutations: HR 3.10 for PFS	Prognostic stratification TP53-mutant may benefit from alternative strategies
Multiple ctDNA Cohorts (FGFR Analysis)	Retrospective ctDNA profiling	Post-CDK4/6i populations	FGFR amplifications highly prevalent in resistant disease	FGFR1/2 amplifications (15–41% by ctDNA)	Potentially actionable target FGFR inhibitors under investigation

## Data Availability

No new data were created or analyzed in this study.

## Abbreviations

The following abbreviations are used in this manuscript:

ADC	antibody–drug conjugate
AE	adverse event
AI	aromatase inhibitor
AKT	protein kinase B
ASCO	American Society of Clinical Oncology
AURKA	aurora kinase A
BC	breast cancer
CDK	cyclin-dependent kinase
CDK2	cyclin-dependent kinase 2
CDK4/6	cyclin-dependent kinase 4 and 6
CDK4/6i	cyclin-dependent kinase 4/6 inhibitor
CCNE	cyclin E
CI	confidence interval
CNS	central nervous system
CT	chemotherapy
ctDNA	circulating tumor DNA
DCR	disease control rate
DNA	deoxyribonucleic acid
D/C	discontinuation
ddPCR	droplet digital polymerase chain reaction
ER	estrogen receptor
ERBB2	Erb-B2 receptor tyrosine kinase 2
ESMO	European Society for Medical Oncology
ESR1	estrogen receptor 1 gene
ET	endocrine therapy
FDA	Food and Drug Administration
FGFR	fibroblast growth factor receptor
HR	hazard ratio
HR+	hormone receptor-positive
HER2	human epidermal growth factor receptor 2
HER2−	human epidermal growth factor receptor 2-negative
ITT	intention-to-treat
mBC	metastatic breast cancer
mPFS	median progression-free survival
mTOR	mechanistic target of rapamycin
NCCN	National Comprehensive Cancer Network
NCT	National Clinical Trial identifier
NDA	new drug application
NGS	next-generation sequencing
OS	overall survival
PD	progressive disease
PDUFA	Prescription Drug User Fee Act
PFS	progression-free survival
PI3K	phosphoinositide 3-kinase
PIK3CA	phosphatidylinositol-4,5-bisphosphate 3-kinase catalytic subunit alpha
PRO	patient-reported outcome
PROTAC	proteolysis-targeting chimera
QoL	quality of life
RAS	rat sarcoma viral oncogene
RB1	retinoblastoma 1 gene
RECIST	Response Evaluation Criteria in Solid Tumors
RWE	real-world evidence
SERD	selective estrogen receptor degrader
SOC	standard of care
TP53	tumor protein 53
TTNT	time to next treatment
VAF	variant allele frequency
